# Multivariate Joint Analysis of Blood Pressure Measurements and Time to Remission: A Case Study of Hypertensive Patients Receiving Treatment at Jimma University Medical Center

**DOI:** 10.34172/jrhs.2025.172

**Published:** 2024-12-25

**Authors:** Jiregna Abebe Akasa, Sisay Wondaya, Shiferaw Befikadu

**Affiliations:** ^1^Department of Statistics, College of Natural and Computational Sciences, Dambi Dollo University, Dambi Dollo, Ethiopia; ^2^Department of Statistics, College of Natural Sciences, Jimma University, Jimma, Ethiopia

**Keywords:** Remission, Systolic and diastolic blood pressure, Longitudinal change, Ethiopia

## Abstract

**Background:** Hypertension (HTN) elevates blood pressure (BP) in the arteries. It is defined as systolic BP (SBP)>140 mm Hg and/or diastolic BP (DBP)>90 mm Hg. This study aimed to identify determinant risk factors of longitudinal change of SBP and DBP with time to first remission of hypertensive patients.

**Study Design:** A retrospective cohort study.

**Methods:** A descriptive and inferential analysis was employed to explore the determinant risk factors, and a multivariate joint model was applied to test the significant association of the possible risk factors.

**Results:** Of all 369 patients, 235 (63.7%) had first remission with a median survival time of five months. The patients demonstrated shorter first remission time when they had no history of comorbidity, resided in urban areas, took a combination of drugs, and were younger. Similarly, residence, age, treatment, history of diabetes mellitus (DM), history of stroke, and observation time were determinant risk factors of SBP. On the other hand, age, treatment, history of DM, chronic kidney diseases, and observation time were identified as determinant risk factors of DBP. The result revealed a strong positive association between changes in SBP and DBP (*P*=0.9923). In addition, a significant association was observed between the value of SBP and time to first remission (γ_1=-0.0693, HR=0.993).

**Conclusion:** Having good follow-ups, receiving control of comorbidity, and taking a combination of drugs show several opportunities for decreasing BP. Consequently, this compels patients to experience the first remission early.

## Background

 Hypertension (HTN) is a chronic medical condition in which blood pressure (BP) is elevated in arteries.^1,2^ In BP measurements, HTN is defined as systolic BP (SBP) equal to or above 140 mm Hg and/or diastolic BP (DBP) equal to or above 90 mm Hg.^3^ Normal levels of both SBP and DBP are particularly important for the efficient function of vital organs.^4^

 HTN is a worldwide public health challenge for both economically developed and developing countries as a leading flexible risk factor for cardiovascular diseases (CVDs) and death. It is becoming more common all over the world and affected 972 million individuals in 2000, with a prevalence rate of 26.4%. In 2025, the number of people affected by HTN is expected to rise to 1.54 billion, with a prevalence rate of 29.2%.^5^ In addition, it is more common in low- and middle-income countries than high-income countries, such as South and East Asia and Sub-Saharan Africa, where 23% of the 1.13 billion adults with high BP lived in South Asia (199 million in India) and another 21% (235 million) lived in East Asia, and there are also more people affected because those countries have more people than high-income countries.^6^

 In Africa, HTN is the most common cause of CVD.^7^ The studies conducted in Ethiopia, Kenya, Nigeria, and Tanzania revealed that the prevalence of HTN in Sub-Saharan African countries ranged from 10.1% in Southern Ethiopia to 23.7% in Tanzania.^8-11^ The prevalence of uncontrolled HTN was high in Ethiopia.^12^ According to data from Ethiopia’s Federal Ministry of Health, HTN was the seventh greatest cause of mortality in 2015, accounting for 1.8% of all deaths.^13^ Based on the evidence from the national non-communicable disease survey of Ethiopia, there was a 15% overall prevalence of HTN.^14^ In some places of southern Ethiopia, the prevalence of HTN ranged from 22% to 35%.^15,16^

 The BP is one of the repeatedly measured statuses over time for hypertensive patient diagnosis. This type of study allows joint modeling of multivariate longitudinal profiles, which is necessary for association structure and evolution of associations, and improves the results of a discriminant analysis of repeatedly measured outcomes.^17^

 Longitudinal and survival data are frequently found combined and are crucial indicators of health in many medical investigations. Repeated measurements of HTN patients taken at various times provide longitudinal observations such as SBP and DBP. The time it takes for each patient to experience the event throughout a certain study period is time-to-event data. Therefore, to simultaneously incorporate all available data and make the best prediction, a joint model (JM) approach is feasible.^18^

 Many well-established approaches exist, including the linear mixed-effects model (LMM) for longitudinal data and parametric or semi-parametric models for survival data separately. The JM, on the other hand, simultaneously estimates both the longitudinal and time-to-event components, making it better suited for evaluating such data because it calculates the relative risk of the time-to-event result based on the longitudinal outcome.^19,20^ When compared to longitudinal and survival models independently, JM allows for significant improvements in estimation accuracy and provides effective and efficient predictions, making accurate uncovering insights.^21^

 Many earlier strategies for combining longitudinal and time-to-event outcomes only allowed for one longitudinal outcome and one event time. However, JM of multiple longitudinal outcomes and a time-to-event, which is called multivariate JM (MVJM), are more beneficial in parameter estimation and result in accurate model predictions.^22^

 Most studies in Ethiopia are focused on longitudinal changes in SBP and DBP over time and separate survival analyses to assess time for good control of HTN.^13,23,24^ However, patients are repeatedly measuring their SBP and DBP, which are potentially predictive for the time-to-event. Concerning the interdependency of these BP outcomes (SBP and DBP) with the time to event, it is necessary to evaluate factors that affect the rate of change in these outcomes jointly.^4^

 As a result, in this study, an MVJM was used, which includes the influence of variables on the time to first remission in the presence of change in BP (SBP and DBP) and an association between the time to remission and BP measurement. This study sought to identify determinant risk factors of longitudinal changes in SBP and DBP with time to first remission of hypertensive patients jointly and to investigate the associations of the evolution of BP measurements over time.

## Methods

###  Study area

 This study was conducted at Jimma University Medical Center (JUMC), which is located in Oromia Regional State, 352 kilometers southwest of Addis Ababa, in Jimma town.^25^

###  Study design and data collection 

 A retrospective study design was employed, and the data were collected from hypertensive patients undergoing treatment at JUMC. The required data were extracted from the medical chart of patients receiving follow-ups (at least three visits) from September 1, 2018, to August 30, 2021.

###  Populations of the study

 The study participants included all patients in JUMC and those who had recorded information on HTN follow-ups. A total of 369 patients were considered in this study based on the inclusion criteria.

###  Variables of the study

 The response variables were the patient’s BP measurements (SBP and DBP) in mm Hg and the time to first remission that it takes to have controlled BP (in months). The day that the patients with HTN were first admitted to the hospital served as the starting point. The end is when the hypertensive patients’ status is back to the normal range of BP or remission (SBP < 140 mm Hg and DBP < 90 mm Hg, respectively) while receiving treatment at the hospital. The observation time, gender, residence, baseline age, regimen type, blood urea nitrogen (BUN), treatment, comorbidity, and serum creatinine level of the hypertensive patients were all the explanatory variables considered in this investigation.

 In this study, if the patients did not experience the event of interest (first remission) up to the end of the study, for the sake of loss to follow up or transfer to another hospital or death due to different causes or even death due to HTN or termination of the study, they all were considered censored.

###  Operational definitions 

*Remission *is defined as if the patients’ BP remains within the normotensive range (SBP < 140 mm Hg and/or BP < 90 mm Hg, respectively) while receiving treatment at the hospital.

*Event* is defined as the first remission of HTN during the follow-up time from September 1, 2018, to August 30, 2021.

*Time to Remission* is the time from the first day of admission for HTN treatment to the first remission.

###  Inclusion and exclusion criteria 

 The inclusion criteria were all hypertensive outpatients, whose age was 18 and above years, regardless of their treatment category during the study period in the hospital, and those outpatients having more than two visit times were included in this study.

 However, hypertensive outpatients admitted with only less than three visits and those under the age of 18 were not included in this study. Additionally, patients with isolated systolic HTN defined as elevated SBP ( ≥ 140 mm Hg) and low DBP ( < 90 mm Hg), isolated diastolic HTN (DBP ≥ 90 mm Hg and SBP < 140 mm Hg), and incomplete recording of baseline were not included in this study.

###  Statistical methods

 In this study, the data were explored by using a Kaplan-Meier curve plot, an individual plot, and a mean profile plot. Then, the time to first remission of HTN and longitudinal measures of SBP and DBP of the patients were taken during the follow-up and separately and jointly analyzed to identify the determinant risk factors. The multivariate joint longitudinal-survival model was analyzed to assess the influence of longitudinal changes in SBP and DBP over time on the first remission of HTN.

 An MVJM is comprised of a multivariate longitudinal data model and a time-to-event data model. Now, let us show the notation for bivariate longitudinal and time-to-event data.

 Let $Y_{ik}\left(t_{ijk}\right)$ denotes the *j*^th^ observed value of the *k*^th^ longitudinal outcome for subject *i*, measured at time *t*_ijk_ for *i* = 1,…,*N*; *k* = 1,...,*K*, and j = 1,..., *n*_ik_. A bivariate linear mixed model (MLMM) is a common approach where measurements for different outcomes can be recorded at different times between patients and outcomes, and it is given by:


(1) $$Y_{ik}\left(t_{ijk}\right)=X_{ik}^{T}\left(t_{ijk}\right)\beta_{k}+Z_{ik}^{T}\left(t_{ijk}\right)b_{ik}+\varepsilon_{ik}$$

Where $X_{ik}^{T}\left(t_{ijk}\right)$ and $Z_{ik}^{T}\left(t_{ijk}\right)$ are row vectors of the covariate for subject *i*, associated with fixed and random effects, respectively, which can vary by outcome; *β*_k_ is a vector of fixed effect parameters for the *k*^th^ outcome, and *b*_ik_ is a vector of subject-specific random effects for the outcome. The vector of subject-specific random effects for all *K* outcomes is denoted by $\;b_{i}={\left(b_{i1},b_{i2},\ldots ,b_{iK}\right)}^{T}\sim N\left(0,\psi \right)$ .

 The *ε*_ik_ represents a corresponding measurement error term such that *ε*_ik_ ~N(0,Σ). In this study, the variance-covariance matrix of random effects ψ implies the correlations among different longitudinal processes as well as the within-subject correlation for each longitudinal measurement (SBP and DBP). The measurement errors of distinct longitudinal outcomes are assumed to be independent of one another, as well as the random effects *b*_i_.^26^

 For survival outcomes, we consider the Cox proportional hazard model,^21^ which is given as:


(2) $$h_{i}\left(t\right)=h_{0}\left(t\right)\exp\left(\alpha_{i}^{T}X_{i}\left(t\right)+\gamma_{i}\left(t\right)\right)$$

Where *X*_i_ is the vector of baseline covariates with the corresponding parameter estimates, and *α*_i_; *h*_0_(*t*) denotes the baseline hazard function. In addition, *γ*_i_(*t*) is the latent process that captures the association structure between the longitudinal measurement and event processes.

###  Model selection and diagnostics 

 The Akaike information criterion (AIC) was used to select the appropriate model, which is a lower criterion value and suggests a better fit. The AIC is given by:


(3) $$AIC=-2logL\left(D;\hat{\theta }\right)+2k$$

Where *k* is the number of parameters, and *n* is the number of observations, and $L\left(D;\hat{\theta }\right)$ is the joint maximized value of the likelihood function of the model, in which $\hat{\theta }\;$ is the parameter value that maximizes the likelihood function. The adequacy of the model, subject-specific residuals, and the marginal residuals were used to assess the assumptions of the standard LMM. The marginal residuals predict the marginal errors and can be utilized to investigate the misspecification of the mean structure and to validate the assumptions for the within-subject covariance structure. By the way, the unstructured covariance structure was selected for LMM.^21^ For survival models, the hazard function of one individual is proportional to that of the other individual (i.e., the hazard ratio is constant over time). The Schottenfeld residuals^27^ and global test of the proportional assumption were employed for this purpose.^28^ The convergence of Monte-Carlo expectation maximization was checked for the adequacy of MVJM; by default, it is checked by the trace plots. The joineRML packages of R-software, which is developed for MVJMs under a classical approach, were used for analysis.^26^

## Results

 This study included a sample of 369 hypertensive patients. The data were explored by using graphical and tabular presentations. Of all patients, 201 (54.5%) were male, and 190 (51.5%) were from urban areas. In general, 235 (63.7%) hypertensive patients treated at the JUMC attained the event of interest (the first remission). Based on the history of other comorbidity diseases, among the hypertensive patients, 51 (13.8%), 68 (18.4%), 241 (65.3%), and 65 (17.6%) had a history of diabetes mellitus (DM), CVD, stroke, and chronic kidney disease (CKD), respectively. Concerning the regimen, about 140 (37.9%), 193 (52.3%), and 36 (9.8%) patients were treated with monotherapy, two therapies, and three or more therapies, respectively. Furthermore, 68 (18.4%), 57 (15.4%), 178 (48%), and 66 (18.2%) patients took amlodipine, enalapril, a combination of amlodipine and enalapril, and other drugs, respectively (Table 1). About 235 (63.7%) hypertensive patients treated at the JUMC attained the event of interest (the first remission). During the follow-up period, 135 (36.6%) of the 235 hypertensive patients having the first remission were male, and 105 (28.5%) of them resided in the urban area. History of CVD and CKD and residence were significantly associated with the time to the first remission of hypertensive patients.

**Table 1:** Frequency distribution of variables with survival status and their association

**Variables**	**Censored (n=134)**		**Event (n=235)**		**Total (n=369)**		***P *** **value**
	**Number**	**Percent**	**Number**	**Percent**	**Number**	**Percent**	
Gender							
Female	68	18.40	100	27.5	168	45.5	0.158
Male	66	17.9	135	36.6	201	54.5	
Residence							
Urban	85	23.0	105	28.5	190	51.5	0.001
Rural	49	13.3	130	35.2	179	48.5	
Diabetes mellitus							
No	111	30.1	207	56.1	318	86.2	0.212
Yes	23	6.2	28	7.6	51	13.8	
Cardiovascular disease							
No	119	32.3	182	49.3	301	81.6	0.010
Yes	15	4.0	53	14.4	68	18.4	
Stroke							
No	41	11.1	87	23.6	128	34.7	0.257
Yes	93	25.2	148	40.1	241	65.3	
Chronic kidney disease							
No	125	33.9	179	48.5	304	82.4	0.001
Yes	9	2.4	56	15.2	65	17.6	
Regimen							
Monotherapy	55	14.9	85	23.0	140	37.9	0.497
Two therapies	63	17.0	130	35.3	193	52.3	
Three or more therapies	16	4.4	20	5.4	36	9.8	
Treatment							
Amlodipine	30	8.4	38	10.0	68	18.4	0.062
Enalapril	21	5.7	36	9.7	57	15.4	
Combinations	68	18.2	110	29.8	178	48.0	

*Source*. Jimma University Medical Center, Ethiopia; the study was performed from 1^st^ September 2018 to 30^th^ August 2021.

 The average SBP and DBP of hypertensive patients were 157.2 (SD = 20.6) mm Hg and 99.6 (SD = 11.7) mm Hg, respectively. The median remission time to have the first remission of hypertensive patients was 5 months. The average creatinine and BUN levels of hypertensive outpatients were 2.2 (SD = 2.1) and 69.2 (55.4) in mg/dL, respectively. In addition, as regards age, it was 47.9 (SD = 16.4) years.

**Figure 1:**
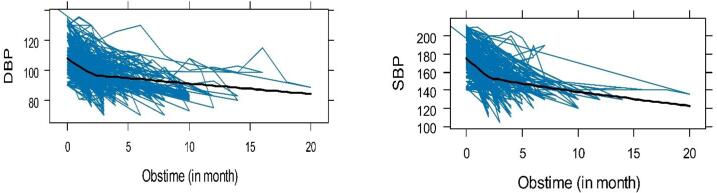
Individual profile plot with average trend line of systolic and diastolic blood pressure

Figure 1 visualizes the individual plots of DBP and SBP. Based on the plot, there was a moderate status within and between individual variations of both outcome measurements over the observation time, indicating that patients began with both varying baselines and different evolutions over time. It also demonstrates the average trend line of both outcome measures, confirming a decreasing trend for both outcomes.

**Figure 2:**
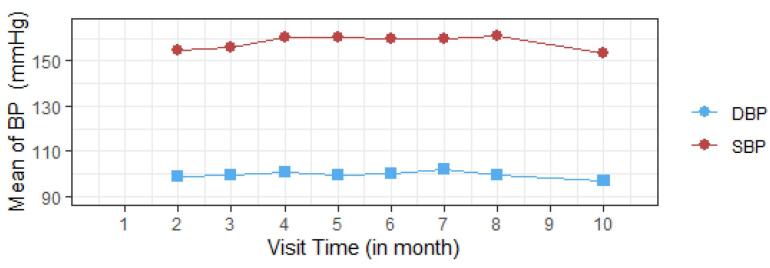
Mean profile plot of systolic and diastolic blood pressure

 The mean progress of both SBP and DBP is shown in Figure 2. This picturized progress of both BP indicates that they have constant changes or progress throughout the visit time. The average of SBP varies between 150 mm Hg and 160 mm Hg, whereas DBP varies between 90 mm Hg and 105 mm Hg.

**Figure 3:**
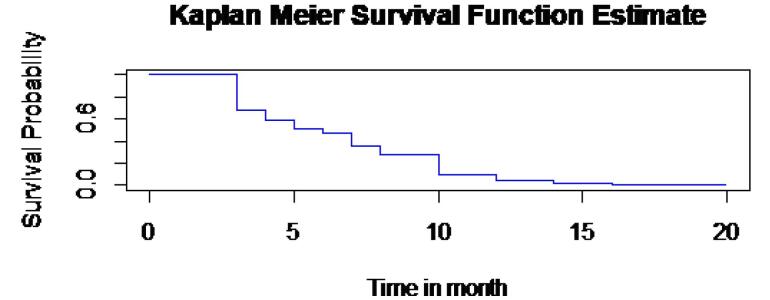
The overall estimate of Kaplan-Meier survivor function plot

 Furthermore, the plot of the Kaplan-Meier estimate of the survivor function is a step function (Figure 3), in which the estimated time to first remission probabilities is constant between adjacent event times but it decreases at each event time. It implies that as a patient’s survival time increases, the probability that hypertensive patients get their first remission decreases over time.

**Figure 4:**
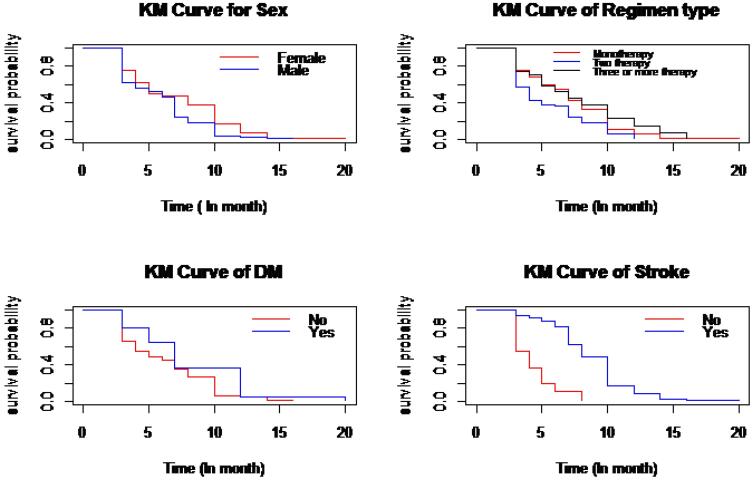
Plot of Kaplan Meier survival curves

 Similarly, the time to first remission of the patients was described using the Kaplan–Meier survival curves (Figure 4).

**Figure 5:**
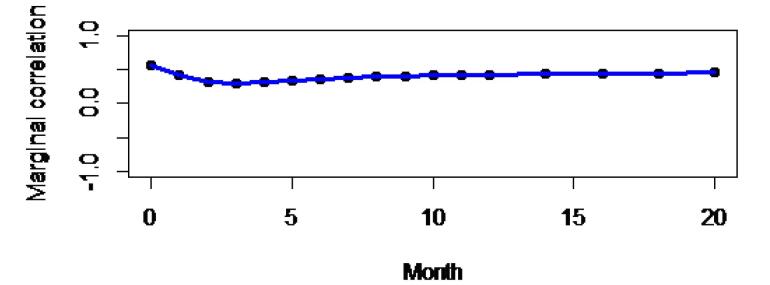
Evolutions of associations

 The result of the variance-covariance matrix from SAS PROC MIXED shows that the variability is higher for SBP (random slope = 30.6981) than for DBP (random slope = 10.6804), indicating that SBP is more variable than DBP over time. Additionally, a high correlation was found between the random intercept of SBP and DBP (0.8588), implying that a patient with a higher initial SBP is likely to have a higher initial DBP. In addition, the association of evolution of SBP and DBP was 0.9923; this suggests that there has been a strong positive association between changes in SBP and DBP over time, and the evolution of association was determined using the marginal correlation between two responses, SBP and DBP, at different visit times. This result is depicted by plotting the marginal correlation over the observation time (Figure 5). This result demonstrates that the evolution of the association of BP levels in hypertensive patients is slightly incremental after the third visit.

 Univariable and multivariable analyses were applied to fit MVJM of BP with time to first remission. In univariable analysis, the model containing each covariate at a time was fitted to determine variables that have the potential for being included in the multivariable analysis. Covariates in the univariable analysis with *P* values less than 25% were considered for multi-variable analysis.

 In univariable analysis, covariates such as gender, comorbidity (CKD, stroke, and DM), baseline age of the patient, BUN level, residence, treatment that patient took, and observation time were included in the linear model of SBP, and for DBP, covariates such as treatment, residence, gender, comorbidity (DM and stroke), BUN level, and observation time were significant at a 25% level of significance. However, the other covariates (e.g., CVD and serum creatinine level) were not significant at the 25% level of significance, and thus they were excluded from the multivariable analysis. Similarly, covariates such as baseline age of the patients, residence, gender, and comorbidity (CKD, DM, stroke, and CVD) were significant at a 25% level of significance and were included in MVJM, whereas others were excluded from the investigation.

 The MVJM explicitly links BP and time-to-first remission processes through the current value association structures parameterization, simultaneously providing a measure of the strength of the association between changes in BPs and the time-to-first remission process. The “mjoint ()” function was applied under the joineRML package to extract the necessary information for MVJM analysis.

 The dataset was fitted with only random intercept and with random intercept and slope under an MVJM. The model with only random intercept had an AIC value of 27233.33, whereas the one with random intercept and slope had a 27024 AIC value. As a result, the most appropriate model is the MVJM fitted with random intercept and slope, which has the lowest AIC value.

 The finding revealed that the average SBP and DBP of patients had a 4.4021 mm Hg and 3.0757 mm Hg increment for hypertensive patients who had a history of DM than those patients with no history of DM. Those patients who were taking a combination of enalapril and amlodipine showed a 5.4088 (SE = 2.1581, *P* = 0.0122) mm Hg decrement in SBP compared to those who were taking a single amlodipine once daily. Similarly, as the patients’ BUN levels increased by a unit mg/dL, the average SBP and DBP increased by 0.0316 mm Hg (SE = 0.0159) and 0.0208 mm Hg (SE = 0.0096), respectively (Table 2).

**Table 2:** Multivariate joint model parameter estimates for longitudinal and survival processes

**Longitudinal Sub-model**					
**Variables**	**Estimate value**	**SE**	***P *** **value**	**95% CI of estimate value**	
Intercept	160.0960	3.2660	0.0001	153.6940	166.4970
Treatment (Enalapril)	1.4086	2.6107	0.5895	-3.7082	6.5255
Treatment (Combination)	-5.4088	2.1581	0.0122	-9.6386	-1.1791
Treatment (Others)	-0.9401	2.3909	0.6942	-5.6260	3.7460
Gender (Male)	3.9378	1.7367	0.0234	-7.3417	-5.3393
CKD (Yes)	3.8075	1.8540	0.0400	0.1738	7.4412
DM (Yes)	4.4021	2.1822	0.0437	0.1250	8.6792
Stroke (Yes)	9.9962	2.3101	0.0001	5.4686	14.5239
Age (Year)	0.1916	0.0438	0.0001	0.1058	0.2775
Residence (Urban)	5.3566	1.7592	0.0023	1.9086	8.8047
BUN	0.0316	0.0159	0.0469	0.0434	0.0628
Observation time (Obstime)	-8.6962	0.4547	0.0001	-9.5875	-7.8050
**Diastolic blood pressure**					
Intercept	101.2576	1.5729	0.0001	98.1749	104.3400
Treatment (Enalapril)	1.9019	1.6393	0.2460	-1.3109	5.1148
Treatment (Combination)	1.2184	1.4303	0.3943	-1.5850	4.0218
Treatment (Others)	1.8930	1.5830	0.2318	-1.2097	4.9960
Residence (Urban)	1.3397	1.0081	0.1839	-0.6361	3.3156
Gender (Male)	-2.1046	0.9159	0.0216	-3.9000	-0.3095
DM (Yes)	3.0757	1.3138	0.0192	0.5007	5.6508
Stroke (Yes)	4.7947	1.4601	0.0010	1.9330	7.6566
BUN	0.0208	0.0096	0.0314	0.0019	0.0397
Observation time	-2.5437	0.1101	0.0001	-2.7595	-2.3280
**Time-to-event sub-model**					
Age	-0.0092	0.0103	0.3706	-0.0293	0.0109
Residence (Urban)	-0.4938	0.3307	0.1354	-1.1420	0.1544
Gender (Male)	0.5930	0.3252	0.0682	-0.0443	1.2303
CKD (Yes)	-0.5852	0.4329	0.1764	-1.4334	0.2633
CVD (Yes)	-1.0038	0.3958	0.0112	-1.7780	-0.2280
DM (Yes)	-0.7638	0.5407	0.1577	-1.8235	0.2960
Stroke (Yes)	-1.9549	0.3898	0.0001	-2.7190	-1.1910
BUN	-0.0054	0.0032	0.0872	-0.0117	7.9168
γ_1_	-0.0693	0.0161	0.0001	-0.1010	-0.0377
γ_2_	-0.0412	0.0499	0.4093	-0.1390	0.0566
**Random effects**					
**Coefficient**	**Variance**	**SD**	***α***_i1_	***b***_i1_	***α***_i2_
α_i1_	255.060	15.97	1.000	-0.7144	0.6467
b_i1_	18.0120	4.244	-	1.000	-0.1196
α_i2_	36.7320	6.064	-	-	1.000
ϵ_i1_	113.96	10.675	-	-	-
ϵ_i2_	73.462	8.571	-	-	-

*Note*. SE: Standard error; CI: Confidence interval; SD: Standard deviation; CKD: Chronic kidney disease; DM: Diabetes mellitus; CVD: Cardiovascular disease; BUM: Blood urea nitrogen. γ_1_ and γ_2_ are the association parameters for the current true value of SBP and DBP, respectively. The *α*_i_ and *b*_i_ correspond to random intercept and slope.

 The hazard of first remission for patients who had a history of stroke was 0.1416 (HR = exp (–1.9549)) times less likely than that of those patients who had no history of stroke. In other words, the time to attain first remission for those patients who had a history of stroke was 85.84% lower than that of patients who had no history of stroke. Moreover, the probability of having first remission for patients who had a history of CVD was 0.3665 (HR = exp (–1.0038)) less likely than that of patients who had no history of CVD (Table 2). The estimate of the association parameter for the current true value of SBP (*γ*_1_) was -0.0693 (HR = exp (–0.0693) = 0.93); this revealed that there was a 0.93 decrement change in the risk of first remission of patients as the current true value of SBP increases in a unit mm Hg. However, the association parameter for the current true value of DBP (*γ*_2_) was not significant.

## Discussion

 In this study, the joint longitudinal of SBP and DBP was used to evaluate the association between two longitudinal progresses, whereas the MVJM of SBP and DBP with time to first remission was utilized on the datasets of hypertensive patients obtained from JUMC. This statistical method was employed to identify the determinant risk factors of longitudinal changes in BP and time to first remission of hypertensive patients and to evaluate the association between the current true values of BP with time to first remission of the patients.

 The results of the joint bivariate longitudinal model revealed that there was a strong association between BP evolutions; this is in line with the findings of a study conducted at Jimma University, which confirmed a strong association between the evolution of SBP and DBP.^29^ Concerning the evolution of the association of BP measurements, in this study, after the third observation, the evolutions of associations slightly increased, which is consistent with the results of previous studies demonstrating that there was a slight increase in the correlation between DBP and SBP over time.^4,29^

 In the multivariate longitudinal sub-model, the observation time was negatively associated with the average progress of SBP and DBP, implying that the average progress of SBP and DBB decreased with an increase in the patient’s observation time. This report is in line with the report of the study conducted at Jimma University^13^ and Felege Hiwot Referral Hospital.^30^ However, age and SBP of the patients were positively associated, revealing that a one-year increase in age was associated with a normal increase of SBP.^4,13,31,32^

 Concerning the anti-hypertensive drugs that patients took, a combination of amlodipine and enalapril was negatively associated with SBP, indicating that their combination decreased the average SBP of hypertensive patients. This result is consistent with the findings of the study conducted on the combination of therapy in HTN, indicating that effective BP reductions were achieved with combination therapy compared to either of the monotherapies.^33,34^ This is because several factors contribute to HTN, and it may be impractical to regulate BP with a single medication that acts through a single mechanism; thus, combination therapy may be a more practical option.

 Patients with a history of DM and stroke showed an average BP (DBP and SBP) that was higher than that of those without such a history. This finding is consistent with the results of studies performed in the northwest of Ethiopia,^35,36^ southwest Ethiopia,^13^ and China.^37^ Likewise, a history of kidney disease was significantly associated with uncontrolled SBP in HTN patients. The average SBP of patients with a history of CKD increased compared to that of those with no history of CKD, which conforms to the results of studies performed in South Asia and China, which showed that diabetic and kidney disease co-morbidities were associated with uncontrolled HTN.^36,38^

 The patient’s residence was significantly associated with the average SBP of patients but not with the DBP of the patients, which corroborates the findings of a study conducted in the southwest of Ethiopia.^29^ The gender of patients was significantly associated with the BP progress; however, there may be disparities between the study reports and those of other studies. This is due to differences in the study area, the presence of comorbidity, environmental variations, and the physical activity trend of patients.^36,39^

 The current true value association parameter in joint analysis for SBP with time to first remission was significant at the 5% level of significance, indicating that there was an association between the current true value of SBP measurement and the risk of first remission, and the risk of first remission was decreased with a unit increase in the value of SBP measurement. This result is in accordance with that of the study conducted at Arba Minch General Hospital, confirming that there was a strong association between SBP measurement and survival time of HTN patients.^18^

 Finally, as taking the combination of amlodipine and enalapril drugs decreases the BP of patients, it is better to treat patients with the appropriate combination of anti-hypertensive drugs by considering their comorbidity. The advanced MVJM was used in this work, which was a simultaneous analysis when there were more than two longitudinal measurements and a time to event. Despite the strength of this study, there were also significant limitations to this study. There may be more than two remission times for hypertensive patients, which was not considered in this study. Accordingly, in addition to the first remission, future studies should apply the JM, which accounts for multiple longitudinal outcomes with multiple time-to-event outcomes.

**Highlights**

- Overall, 235 (63.7%) hypertensive patients treated at JUMC attained the first remission.
- Patients receiving a combination of anti-hypertensive drugs were more likely to have first remission than those receiving single drugs.
- Time to first remission was correlated with patients’ systolic blood pressure (SBP).
- Patients having comorbidity stayed a long time without having the first remission.

## Conclusion

 Based on the findings, patients receiving a combination of treatments had lower SBP and DBP as compared to others who only received amlodipine anti-hypertensive drugs, which may take the patients as they have first remission within a short period of time. In other words, the risk of having first remission was significantly increased. This justification was observed from the JM of the longitudinal and time-to-event models. On the other hand, having a history of comorbidity could significantly determine the time to first remission of patients. Patients who had a good follow-up and took a combination of drugs have an opportunity to decrease their BP. This makes patients have their first remission within a short period of time than others. In addition, patients’ age and history of stroke, DM, and CKD were the joint determinants of longitudinal changes in BP and the first remission time, which makes patients not to have first remission early. Decreasing BP leads to good control of HTN by all means.

## Acknowledgements

 The authors would gratefully like to acknowledge JUMC for providing data.

## Competing Interests

 The authors declare that there was no conflict of interests in this study.

## Ethical Approval

 Ethical approval was obtained from the Institutional Research Ethics Review Committee of the Jimma University College of Natural Sciences. A letter of support was written to Advanced Healthcare Management Corporation (AHMC). The authors submitted an official letter to AHMC. After clarifying the purposes of the study, the secondary data were obtained from all subjects and/or their legal guardian(s) for the participated cases who were adults above 18 years old. All methods were performed following the relevant guidelines and regulations.

## Funding

 There was no direct fund for this study.
